# Benefits of Fiber-Enriched Foods on Satiety and Parameters of Human Well-Being in Adults with and without Cardiometabolic Risk

**DOI:** 10.3390/nu15183871

**Published:** 2023-09-06

**Authors:** Janine Ehret, Beate Brandl, Karsten Schweikert, Rachel Rennekamp, Nanette Ströbele-Benschop, Thomas Skurk, Hans Hauner

**Affiliations:** 1 Department of Applied Nutritional Psychology, Institute of Nutritional Medicine, University of Hohenheim, 70599 Stuttgart, Germany; 2 ZIEL-Institute for Food and Health, Technical University of Munich, 85354 Freising, Germany; 3 Else Kroener-Fresenius-Centre of Nutritional Medicine, Clinical Nutritional Medicine, TUM School of Life Sciences, Technical University of Munich, 85354 Freising, Germany; 4 Core Facility Hohenheim, University of Hohenheim, 70599 Stuttgart, Germany; 5 Institute of Nutritional Medicine, School of Medicine, Technical University of Munich, 80333 Munich, Germany

**Keywords:** fiber, fiber-enriched foods, quality of life, satiety, well-being

## Abstract

Consumption of fiber-rich foods is linked to beneficial effects on chronic diseases and gut health, while implications towards improving satiety and parameters of well-being remain unclear. A randomized placebo-controlled intervention study was conducted to compare the effects of fiber-enriched foods to their non-enriched counterparts in adults over a 12-week period on selected clinical parameters—satiety, quality of life, body sensation, and life satisfaction—subjective health status, and importance of diet for well-being. Quality of life (QOL) differed significantly between intervention and control groups at baseline, throughout, and at the end of the study. No effects on satiety, satisfaction with life, or the importance of diet for well-being could be shown between groups. With higher fiber intake, body sensation ratings increased. A higher BMI was significantly associated with lower-body sensation, subjective health status and quality of life. Fiber-enriched foods do not seem to affect feeling of satiety or parameters of well-being. Larger samples and additional methods are necessary to fully explore the effect of increased fiber intake on patient-related outcomes in more detail.

## 1. Introduction

Benefits of high fiber and whole grain intake for human health are well known [1], particularly in the prevention and treatment of cardiometabolic diseases such as obesity, type 2 diabetes, or cardiovascular diseases [2,3,4]. There is also scientific evidence for them preventing various cancers and reducing cancer mortality [5,6]. Thus, many national dietary guidelines promote the consumption of fiber-rich foods. Recommendations vary from 25 to ≥35 g fiber intake per day by consuming whole grain cereals and whole grain cereal products as well as by adding more fiber-rich foods, such as legumes, vegetables, fruits, and nuts, to the diet [7,8,9]. Data from the National Consumption Survey 2 show that women fail to reach the above-mentioned range of recommendations on average, while men, due to the higher overall energy intake, reach the lower limit of 25 g/d [10]. Internationally, it is evident that previous strategies to increase dietary fiber intake are not sufficient [11]. A new approach is the enrichment of foods to compensate for nutrient deficiencies, for example, in gluten-free foods. As functional foods continue to gain popularity and acceptance through improved industrial formulations, fiber enrichment of widely consumed foods could help improve fiber intake [11,12]. A recent randomized intervention study which underlies the previously unpublished, additionally collected parameters presented in this short communication compared a diet with fiber-supplemented foods to a control diet over the study duration of 12 weeks to increase fiber intake by 10 g per day [13]. Participants in both groups were of similar age (53 ± 7 years (intervention group) vs. 52 ± 6 years (control group)). The fiber-enriched foods were well accepted, particularly popular convenience foods such as pizza. The results showed an increase in fiber intake from 22.5 ± 8.0 to 36.0 ± 8.9 g/day in the intervention group after 12 weeks, which was significantly more than in the control group, whose fiber intake (24.1 ± 8.7 to 22.9 ± 6.8 g/day) did not change significantly [13].

In addition to the health benefits mentioned above, dietary fiber is also considered to increase satiety. Approaches to improve satiety through appropriate food choices remain relevant due to the high prevalence of overweight and obesity. While oat fiber ([14,15,16,17,18,19] vs. [20,21]), including ß-glucans, psyllium husk fiber [22,23], and powder cellulose [22], seems to increase satiety relatively constantly, tendencies are less clear for inulin ([14,24,25,26,27] vs. [19,21,28,29,30,31,32]) and dextrin ([19,33,34,35] vs. [36,37,38]), and they are not convincing for wheat fiber [18,39,40]. Since most of the fiber-enriched foods as well as three out of four of the most popular food groups contained dietary fiber for which a positive influence on satiety is likely to be assumed, it was hypothesized that satiety would increase in the intervention group of our randomized controlled trial [13].

Fiber-rich foods are known for their risk of causing short-term gastrointestinal complaints such as bloating, but acceptance and tolerance often increase after getting used to a higher fiber intake [20]. This might result in deterioration of body sensation at the beginning of the study and normalization as the investigation progresses. 

Low-fiber diets are associated with an increased prevalence of constipation complaints, and fiber-rich foods, especially foods containing soluble fiber, show potential for reducing the severity of those symptoms. Dietary fiber could, therefore, relieve constipation, consequently improving quality of life (QOL) [41,42]. Results in children and patients with inflammatory bowel syndrome show promising results [43,44,45,46]. 

Regardless of food-related QOL, Ramin et al. studied how fiber intake relates to mental-health-related QOL in postmenopausal women [47]. They found that fiber intake and whole grain consumption significantly correlated with increased QOL. In contrast, Dalile et al. reported no effect of dietary fiber on mood and psychobiological function in healthy men [48]. Borkoles et al. were able to show across all groups (with the exception of the week in which the highest fiber dose was consumed for the first time) that more fiber resulted in higher ratings of “feeling well” and “feeling less tired” as well as the impression of being able to accomplish more in everyday life [20]. However, the control group also showed improved well-being scores. It is assumed that the effects of fiber on cognitive performance are related to changes in the gut microbiota [49]. In the current study, we were interested to study as to whether a fiber-enriched diet may affect ratings of perceived satisfaction with life, importance of food for well-being, body sensation and quality of life in a real-world setting in comparison to a habitual diet without fiber enrichment. 

## 2. Materials and Methods

The study protocol was approved by the Ethical Committee of the Faculty of Medicine of the Technical University of Munich (approval no. 201/17S). All procedures used were in agreement with the Declaration of Helsinki of 1975, as revised in 2013. Written informed consent was obtained from all participants before starting the study procedures. 

Detailed information about study participants, intervention, and design of the Freising Fiber Acceptance Study has been previously reported [13]. The study, with 115 participants, was designed as a single-blinded, randomized, controlled two-arm comparison and covered an intervention and observation period of 12 weeks. Both groups included healthy individuals with normal weight as well as participants with elevated waist circumference (>102 cm in males, >88 cm in females), which was used as an indicator of elevated cardiometabolic risk. Study visits took place at baseline (visit 1), after 4 weeks (visit 2), and after 12 weeks of intervention (visit 3).

Quality of life was assessed using the validated questionnaire EUROHIS-QOL 8 [50], while all other parameters were assessed using self-generated questions shown in Table 1. Five-point Likert scales were chosen as response scales (with the exception of satiety). Estimation of rate of food intake was surveyed as a control parameter for satiety.

Data analysis was performed in R version 4.2.1. Results are represented as mean ± SD. *p* < 0.05 was assumed to be statistically significant. A linear mixed model was used to analyze variables with an interval scale, while a generalized linear mixed model was estimated for ordinally scaled dependent variables. For this purpose, the R packages lme4 and ordinal were utilized. Intervention, cardiometabolic risk, BMI, and fiber intake were assumed to be fixed effects. ID and time of visit were considered to be random effects. 

## 3. Results

Baseline characteristics have already been reported elsewhere [13] and did not significantly alter between the intervention (*n* = 74) and the control group (*n* = 34).

However, quality of life differed significantly between the intervention and control group at baseline and at Visits 2 and 3. Using the full sample and controlling for individual heterogeneity and repeated measurement in a linear mixed model, we could find that the quality of life of the intervention group was on average 1.92 points higher (Visit 1 (baseline): 33.78 ± 2.81 (*n* = 74) vs. 32.18 ± 4.16 (*n* = 34); Visit 2: 33.66 ± 3.12 (*n* = 73) vs. 31.88 ± 3.88 (*n* = 33); Visit 3: 33.86 ± 3.21 (*n* = 74) vs. 32.00 ± 4.24 (*n* = 32), t = −2.79). There was no significant change in quality of life in either group during the 12-week intervention period.

For the feeling of satiety, no difference was found between the intervention and control group at baseline, throughout, and at the end of the study. No significant differences were found between the groups with regard to life satisfaction and the importance of diet and food for well-being; the same applied to body sensation. No differences were found in any variable between subjects with and without increased cardiometabolic risk. A significantly higher body sensation was measured with increasing fiber intake. A higher BMI showed a highly significant association with a lower assessment of body sensation and was significantly associated with a lower subjective current health status (see Table 2).

In addition, a model with the time of visit as a fixed effect was estimated. Here, significant associations were found for satiety and satisfaction with life over the course of the study. Pairwise comparisons were further analyzed using a post-hoc Tukey test in Table 3. For the remaining dependent variables, no significant effects of time of visit were found. 

To examine these findings more closely, another model that considered the interaction between the time of visit and group assignment was estimated. This interaction model did not show significant results regarding the feeling of satiety. 

## 4. Discussion

For quality of life, significantly higher values were reported in the intervention group than in the control group. However, this was already the case at the beginning of the study, so this effect cannot be attributed to the intervention. Looking at the occurrence of gastrointestinal complaints from the previous data analysis [13], no correlation was found. Although the frequency of bloating (from 50 to 55.4%) and flatulence (56.8 to 71.6%) in the intervention group increased over the course of the study, no deterioration in quality of life was measurable. For the control group, in which the frequency of bloating (55.9% to 44.1%) decreased but flatulence (52.9 to 58.8) also increased, no change in quality of life could be measured either. The fact that quality of life did not decrease despite slightly increasing gastrointestinal symptoms can possibly be interpreted as introduction of additional fibers into the usual diet does not be recognized as restrictive. 

Hettich et al. recently revalidated the questionnaire used and published updated reference values for the average German population [51]. It turned out that the measured quality of life of the study participants in our study was, on average, higher at each visit than the average value of 31.66 ± 5.25 collected in 2021 by Hettich et al. According to the authors, this is possibly related to the effects of the COVID-19 pandemic, which did not yet play a role when the intervention study underlying the data was conducted. In addition, both male sex, older age, and higher education level are associated with a higher quality of life [52]. As there were no significant differences in age between the groups, age was not included in the analysis. There was a slightly higher proportion of males in the intervention group than in the control group (45.95% vs. 41.18%), which may have contributed to the differences measured. There may also have been differences in educational level between the groups, which were neglected in the analysis. A negative association between higher body weight and lower quality of life was also found independently of groups. This finding is in line with expectations and has already been shown [53,54]. 

Results on the effect of the intervention on satiety were mixed. No differences were found between the groups. While the first model also found no difference during the intervention, the second model showed a highly significant positive association between baseline and 4 weeks and a significant positive association between baseline and 12 weeks. It is possible that the test sensitivity of the question used was insufficient to show clear results since satiety was not measured after each meal using visual analog scales, as is common [55]. In addition, the real-world setting did not allow the products provided to be consumed in a standardized manner, as recommended for studies investigating effects of dietary fiber on satiety [56]. However, the results of the present study are in line with the majority of studies that investigated the short-term effects of fiber enrichment under more standardized conditions [57]. However, this less strict approach offered the advantage of obtaining results that more accurately reflected the potential benefits of such products in daily life. Studies of similar duration and setting show both concordant [58] and divergent [59] results on hunger and appetite ratings. Compared to many other studies on satiety, the duration of 12 weeks, which is also explicitly recommended in some cases [56], should be positively highlighted. Since satiety may also be related to the matrix of the food consumed [56], the results could have been more differentiated if similarly constituted fiber-enriched foods (e.g., fiber drinks and soup vs. bread, bakery products, and pizza) had been available at different phases of the study. This would, however, have contradicted the study concept. Finally, it is still possible that a restriction of viscous dietary fibers (ß-glucan, psyllium, polydextrose) for fiber enrichment of the provided foods would have led to clearer results [60], but this would have resulted in a smaller range of fiber-enriched products. 

Ambiguous results also emerged for life satisfaction. While no differences were found in the first model, a closer look at the visits in the second model showed highly significant negative associations. Gazek and Wojtowicz reported that an increase in life satisfaction is associated with healthier food choices in women with diabetes [61]. Healthier food choices included high-fiber foods such as vegetables, legumes, and whole grains. Because study participants were informed about the health benefits of fiber but were instructed not to change their food choices, the study design may have prevented a positive effect on life satisfaction, even though fiber intake increased significantly. A worsening of life satisfaction could possibly have been caused by the discrepancy between provided fast-food products and the recommendations for a healthy diet. It is possible that other outcomes could have been measured using a more complex instrument [62]. This can also be assumed to assess the importance of nutrition and food for well-being, which was also examined in a non-validated manner. Again, it might be assumed that the advice not to change food choices during the study could have avoided some effects.

For the assessment of the subjective current health status, there were no differences between the intervention and control group. However, a significant negative correlation with BMI was found, consistent with the negative associations found between BMI and quality of life. Negative influences on self-rated health have been reported in the literature for both very low and extremely high BMI [63]. For other factors influencing self-rated health (e.g., sleep quality, education, income, working hours, and partnership [64]), no change was expected from the intervention, so these were not included. 

There was also no effect on ratings of body sensation. However, similar to the subjective current health status, a highly significant negative association with BMI was measurable. The question used can be understood as a part of body image measurement [65]. Body image has been described as a crucial mediator between increased body weight and emotional well-being [66]. Therefore, it is encouraging that a significant positive association of higher dietary fiber intake with body sensation was measurable independent of the group. Again, the result would be more reliable if a validated questionnaire had been used. The exact causes of this effect cannot be explained by the analysis performed. 

In order to clarify the influence of increased dietary fiber intake on well-being, further studies with more test-sensitive measuring instruments over a longer period of time are desirable. By capturing most parameters with one question, it was possible to capture more parameters of well-being than would have been the case with more comprehensive survey instruments, as the length might have led to incomplete answers. To determine differences between groups, groups of equal size might have been more appropriate. Regarding further subdivision into subjects with and without cardiometabolic risk, the resulting small number of cases might have been a limiting factor, especially in the control group. Overall, the feasibility of a study design for measuring satiety close to everyday life was piloted, some already known relationships between BMI and the measured parameters of well-being could be confirmed, and a promising indication of the influence of dietary fiber on body sensation was found. Whether dietary fiber contributes to improved satiety remains unclear. Further studies are needed to find out to what extent the effects differ between increasing fiber intake from natural sources and fiber-enriched foods.

## Figures and Tables

**Table 1:** Parameters used and survey questions to determine satiety and well-being.

Parameter	Assessment
Satiety	When I eat... (1 = …I’m already full after a few bites, 2 = …I’m full after about a third of the meal, 3 = …I’m full after a good half of the meal, 4 = …I’m full when I’ve eaten most of the meal, 5 = …I hardly ever feel full)
Satisfaction with life	On a scale of 1 to 5, please indicate how satisfied you are with your life in general. (1 = not at all satisfied, 2 = not very satisfied, 3 = neither, 4 = somewhat satisfied, 5 = very satisfied)
Body sensation	Please use a scale to indicate how comfortable you would currently rate your body feeling. (1 = very uncomfortable, 2 = somewhat uncomfortable, 3 = neither, 4 = somewhat comfortable, 5 = very comfortable)
Subjective current health status	Please indicate on a scale how you assess your current health situation. (1 = very bad, 2 = bad, 3 = neither, 4 = good, 5 = very good)
Importance of nutrition and food on well-being	Please use a scale to indicate how important nutrition and food are for your well-being. (1 = very unimportant, 2 = unimportant, 3 = neither, 4 = important, 5 = very important)
Estimation of rate of food intake	How would you describe the rate of your food intake compared to others? (1 = very slow, 2 = relatively slow, 3 = comparable, 4 = relatively fast, 5 = very fast)

**Table 2:** Regression results from generalized linear mixed models.

Fixed Effects	Estimates (SE) ^1^				
	Satiety	Satisfaction with Life	Body Sensation	Health Status	Nutrition and Well-Being
Intervention	0.78 (1.06)	−1.42 (1.07)	−0.65 (0.56)	−0.25 (0.67)	1.33 (0.71)
CMR (cm)	−1.76 (1.67)	0.21 (1.53)	−0.42 (0.81)	−0.20 (0.96)	0.16 (0.98)
BMI (kg/m^2^)	0.22 (0.19)	−0.11 (0.19)	−0.29 ** (0.10)	−0.23 *(0.12)	0.00 (0.12)
Fiber intake (g)	0.02 (0.04)	−0.00 (0.03)	0.04 * (0.02)	0.03 (0.02)	−0.01 (0.02)
Rate of food intake	0.23 (0.42)	-	-	-	-

^1^ Data expressed as parameter estimates, standard error (SE) and *p*-values; *p*-values less than 0.05 are considered significant; * *p* < 0.05; ** *p* < 0.01. Results are from generalized linear mixed models using ID and time of visit as random effects; CMR = cardiometabolic risk (waist circumference > 102 cm in males and >88 cm in females), BMI = body mass index (kg/m^2^), fiber intake (g) = dietary fiber intake in grams, rate of food intake = estimation of rate of food intake, health status = subjective current health status; nutrition and well-being = importance of nutrition and food on well-being.

**Table 3:** Regression results from generalized linear mixed models with visits as fixed effect.

	Estimates (SE) ^1^	
Contrasts	Satisfaction with life	Satiety
Visit 1–Visit 2	−0.02 *** (0.00)	2.15 ** (0.68)
Visit 1–Visit 3	−0.61 *** (0.00)	1.82 * (0.69)
Visit 2–Visit 3	−0.60 *** (0.00)	−0.34 (0.51)

^1^ Data expressed as parameter estimates, standard error (SE) and *p*-values; *p*-values less than 0.05 are considered significant; * *p* < 0.05; ** *p* < 0.01, *** *p* < 0.001. Results are from generalized linear mixed models using ID as random effects; Visit 1 = visit at baseline; Visit 2 = visit after 4 weeks of intervention; Visit 3 = visit after 12 weeks of intervention.

## Data Availability

Source code data are available from the corresponding author upon request.
